# Contribution of cortical lesions to cognitive impairment in Japanese patients with multiple sclerosis

**DOI:** 10.1038/s41598-020-61012-3

**Published:** 2020-03-23

**Authors:** Koji Shinoda, Takuya Matsushita, Yuri Nakamura, Katsuhisa Masaki, Shiori Sakai, Haruka Nomiyama, Osamu Togao, Akio Hiwatashi, Masaaki Niino, Noriko Isobe, Jun-ichi Kira

**Affiliations:** 1 Department of Neurology, Neurological Institute, Graduate School of Medical Sciences, Kyushu University, Fukuoka, Japan; 2 Department of Clinical Radiology, Graduate School of Medical Sciences, Kyushu University, Fukuoka, Japan; 3 Department of Molecular Imaging & Diagnosis, Graduate School of Medical Sciences, Kyushu University, Fukuoka, Japan; 4 Department of Clinical Research, National Hospital Organization Hokkaido Medical Center, Sapporo, Japan; 5 Department of Neurological Therapeutics, Neurological Institute, Graduate School of Medical Sciences, Kyushu University, Fukuoka, Japan

**Keywords:** Multiple sclerosis, Multiple sclerosis

## Abstract

Cortical lesions (CLs) have a low prevalence and are associated with physical disabilities in Japanese patients with multiple sclerosis (MS). However, the contribution of CLs to cognitive impairment remains unclear in Asian MS. Sixty-one prospectively enrolled MS patients underwent three-dimensional double inversion recovery MR imaging, the Brief Repeatable Battery of Neuropsychological Tests (BRB-N), the Apathy Scale (AS), the Fatigue Questionnaire (FQ), and the Hospital Anxiety and Depression Scale (HADS) within a 1-week period. The cognitive impairment index (CII) score was calculated to measure patients’ overall cognitive impairment. MS patients with CLs had poorer scores than those without CLs in most BRB-N tests, but scored comparably in the FQ, AS, and HADS. The number of CLs correlated negatively with all BRB-N test scores and positively with total CII scores. Leukocortical lesions were more extensively associated with cognitive dysfunction in various domains than intracortical lesions. Stepwise multiple regression analysis revealed that potential confounding factors for the highest quartile of CII score were the number of CLs (odds ratio 2.38, *p* = 0.0070) and the Expanded Disability Severity Scale score (odds ratio 2.13, *p* = 0.0003). Our results demonstrate that the presence and number of CLs are robustly associated with cognitive dysfunction in Asian MS patients.

## Introduction

Multiple sclerosis (MS) is an autoimmune demyelinating disease of the central nervous system that affects not only motor, but also cognitive and psychiatric functions. Cognitive impairment has been reported in 43–70% of MS patients^1–3^ and increases the risk of loss of employment or reduction in vocational duties^4^. In MS, information processing speed, visual memory, visuospatial perception, and working memory are particularly impaired from the early phase of the disease^1^. No large-scale epidemiological studies have investigated the prevalence of cognitive impairment among Japanese patients with MS. However, in a small case series, attention deficits characterized by slow automatic information processing, a very early sign of cognitive impairment in MS, were found in 14 of 22 (66.7%) patients^5^. Several studies have also reported significantly lower scores in the cognition test batteries, including the Brief Repeatable Battery of Neuropsychological Tests (BRB-N) used in the present study, among Japanese MS patients compared with healthy controls^6–9^.

The advent of new magnetic resonance imaging (MR) sequences, such as double inversion recovery (DIR) and phase-sensitive inversion recovery, have facilitated the identification of cortical lesions (CLs) in patients with MS, even in the early phase of the disease^10^. In Caucasians, there are several lines of evidence demonstrating that CLs contribute to cognitive dysfunction in patients with MS^11–15^. Cognitively impaired relapsing-remitting MS (RRMS) patients have higher numbers and greater volumes of intracortical lesions (ICLs) compared with cognitively normal MS patients^11^. Prospective studies have shown that CLs accumulate over time in association with cognitive decline^12,13^. One study reported that leukocortical lesions (LCLs) affecting both grey matter (GM) and white matter (WM) make a greater contribution to cognitive impairment than ICLs in MS^13^. Recent 7-tesla MRI studies have reported that LCLs are more extensively associated with various domains of cognitive impairment than ICLs^14,15^.

Despite the serious impact of cognitive decline on the quality of life of patients with MS, only a few studies have investigated cognitive impairment and CLs in Asian patients with MS^16,17^, who present features distinct from those of European patients with MS^18,19^. We recently reported that CLs were found at a relatively low frequency (29.7%) on DIR imaging of 3-tesla MRI among Japanese patients with RRMS^16^ compared with that among Caucasian patients with RRMS (≥ 60%)^20–23^. Moreover, the number of CLs was significantly associated with motor disability in Japanese patients^16^. Furthermore, because two human leukocyte antigen (HLA) risk alleles, HLA–DRB1*15:01 and HLA–DRB1*04:05, are prevalent in Japanese MS patients^24^, we also studied the influence of these HLA alleles on CLs and found that HLA–DRB1*15:01 increases CLs, whereas HLA–DRB1*04:05 decreases CLs^16^. This finding is consistent with the result from a previous pathological study, which found that HLA–DRB1*15:01 was associated with greater cortical inflammation in autopsied MS materials^25^, suggesting an involvement of HLA class II molecules in CL development^26,27^. Another small-scale study of Japanese MS patients that assessed cognitive function reported that the attention of 13 CL-positive patients was significantly worse than that of six CL-negative patients; however, the study did not classify CLs into ICLs and LCLs and did not evaluate the relationship between CL quantity and cognitive function^17^.

Therefore, it is possible that despite the lower quantity of CLs in Japanese MS patients compared with Caucasian MS patients, CLs may have similar effects on cognitive function in Japanese and Caucasian MS patients. The effects of different CL subtypes, particularly ICLs and LCLs, on cognitive dysfunction remains to be elucidated in Asian patients, including Japanese patients. Therefore, we aimed to elucidate the effects of CLs on cognitive functions by using a comprehensive, prospective assessment of these functions, combined with DIR neuroimaging. In particular, our focus was the differential effects of ICLs and LCLs on cognition and the association between the *HLA–DRB1* genotype and cognitive impairment in Japanese patients with MS.

## Results

### Demographics

The participants consisted of 61 MS patients in the remission phase (42 RRMS and 19 secondary progressive MS [SPMS]) (Table 1). Mean disease duration was 13.5 ± 10.5 years. CLs, ICLs, and LCLs were observed in 27 (44.3%), 13 (21.3%), and 25 patients (41.0%), respectively. Among the 27 MS patients with CLs, 11 (40.7%) had both LCLs and ICLs, 14 (51.9%) had only LCLs, and two (7.4%) had only ICLs. The numbers of CLs, ICLs, and LCLs (mean ± SD) were 1.31 ± 2.54, 0.31 ± 0.65, and 1.01 ± 2.11, respectively (Table 1).

**Table 1:** Clinical demographics and laboratory results of the participants.

	HCs (n = 115)	MS (n = 61)	*p* value
Sex, female	91 (79.8%)	51 (83.6%)	NS
Age at examination, years	40.3 (10.3)	43.0 (11.7)	NS
Education, years after 15 years old	4.8 (1.8)	4.7 (2.0)	NS
Age at disease onset, years	—	29.4 (10.6)	—
Disease duration, years	—	13.5 (10.5)	—
RRMS/SPMS	—	42 (68.9%)/19 (31.1%)	—
Annualized relapse rate^†^	—	0.44 (0.52)	—
EDSS score	—	3.14 (2.50)	—
MSSS	—	3.67 (2.92)	—
Oligoclonal IgG bands	–	32/51 (62.8%)	—
IgG index^††^	—	0.83 (0.44)	—
Number of CLs	—	1.31 (2.54)	—
Number of ICLs	—	0.31 (0.65)	—
Number of LCLs	—	1.01 (2.11)	—
Treatment	—	Fingolimod (n = 24), Interferon β-1a (n = 11), Natalizumab (n = 4), Interferon β-1b (n = 3), Azathioprine (n = 2), Glatiramer acetate (n = 1), None (n = 16)	—

Data are shown as the mean (standard deviation) or n (%). NS, not significant. ^†^Data of the annualized relapse rate was available for 53 patients. ^††^IgG index was examined in 50 patients.

### Neuropsychological assessment of MS patients with and without CLs

We first compared our MS patients with age- and sex-matched healthy controls previously subjected to BRB-N who were included in our previous study^6^. Overall, MS patients showed significantly lower scores than controls in the following tests (Supplementary Table 1): SRT-LTS (*p* = 0.0367), SPART (*p* < 0.0001), SPART-D (*p* = 0.0169), SDMT (*p* < 0.0001), PASAT-3 (*p* < 0.0001), PASAT-2 (*p* < 0.0001), and the WLG test (*p* < 0.0001).

Japanese MS patients with CLs had significantly lower scores than those without CLs in the following tests (Table 2): SRT-LTS (*p* = 0.0201), SPART (*p* = 0.0335), SPART-D (*p* = 0.0082), SDMT (*p* = 0.0080), PASAT-3 (*p* = 0.0033), and PASAT-2 (*p* = 0.0042). Similarly, CII scores were significantly poorer in MS patients with CLs than in those without CLs (*p* = 0.0019). However, there were no significant differences in AS, FQ, HADS-A, or HADS-D scores between MS patients with and without CLs.

**Table 2:** Neuropsychological assessments of MS patients with or without CLs, ICLs, or LCLs.

	CLs (+) (n = 27)	CLs (−) (n = 34)	*p* value	ICLs (+) (n = 13)	ICLs (−) (n = 48)	*p* value	LCLs (+) (n = 25)	LCLs (−) (n = 36)	*p* value
SRT-LTS	38.6 (17.2)	48.0 (15.2)	0.0201	37.2 (22.3)	45.6 (14.5)	NS	38.4 (17.4)	47.6 (15.2)	0.0262
SRT-CLTR	32.7 (18.7)	41.8 (17.3)	0.0589	33.2 (23.6)	39.0 (16.8)	NS	32.3 (18.9)	41.6 (17.2)	0.0565
SRT-D	8.04 (3.74)	9.62 (2.76)	NS	7.38 (4.50)	9.33 (2.81)	NS	8.08 (3.75)	9.50 (2.85)	NS
SPART	18.1 (4.93)	20.6 (5.43)	0.0335	15.7 (5.06)	20.5 (4.95)	0.0043	17.8 (4.92)	20.7 (5.34)	0.0179
SPART-D	6.30 (2.49)	7.94 (1.97)	0.0082	5.92 (2.53)	7.56 (2.19)	0.0325	6.20 (2.53)	7.92 (1.95)	0.0072
SDMT	38.5 (14.1)	48.6 (14.1)	0.0080	37.5 (16.0)	45.9 (14.2)	0.0891	38.0 (14.5)	48.3 (13.8)	0.0077
PASAT-3	36.9 (12.9)	46.1 (10.8)	0.0033	32.8 (14.7)	44.5 (10.8)	0.0117	36.4 (12.9)	45.9 (10.9)	0.0020
PASAT-2	26.4 (10.0)	33.6 (10.1)	0.0042	23.2 (9.87)	32.4 (10.0)	0.0075	26.4 (10.3)	33.2 (10.0)	0.0084
WLG	21.3 (7.27)	24.6 (6.26)	0.0959	18.5 (7.43)	24.4 (6.20)	0.0126	21.0 (7.34)	24.6 (6.20)	0.0686
CII	8.11 (5.47)	4.00 (4.38)	0.0019	9.85 (6.19)	4.73 (4.47)	0.0065	8.12 (5.42)	4.22 (4.59)	0.0033
AS	16.9 (7.91)	15.1 (6.94)	NS	18.3 (8.82)	15.2 (6.89)	NS	16.0 (7.47)	15.8 (7.41)	NS
FQ	123.7 (33.8)	124.4 (28.6)	NS	116.1 (33.8)	126.3 (29.9)	NS	124.3 (35.1)	124.0 (27.9)	NS
HADS-A	6.07 (3.14)	7.97 (4.48)	NS	5.85 (3.13)	7.48 (4.20)	NS	5.96 (3.23)	7.94 (4.36)	NS
HADS-D	7.11 (3.48)	7.03 (5.11)	NS	7.15 (3.95)	7.04 (4.58)	NS	6.84 (3.47)	7.22 (5.03)	NS

Data are shown as the mean (standard deviation). NS, not significant.

MS patients with ICLs had significantly lower scores on the SPART (*p* = 0.0043), SPART-D (*p* = 0.0325), PASAT-3 (*p* = 0.0117), PASAT-2 (*p* = 0.0075), and WLG test (*p* = 0.0126) and higher CII scores (*p* = 0.0065) than those without ICLs (Table 2). MS patients with LCLs had significantly lower scores on the SRT-LTS (*p* = 0.0262), SPART (*p* = 0.0179), SPART-D (*p* = 0.0072), SDMT (*p* = 0.0077), PASAT-3 (*p* = 0.0020), and PASAT-2 (*p* = 0.0084) and higher CII scores (*p* = 0.0033) than those without LCLs (Table 2). Subsequently, among MS patients with CLs, we compared patients who had ICLs with those who did not have ICLs. MS patients with ICLs had significantly lower SPART scores (mean ± SD: 15.7 ± 5.1 vs. 20.4 ± 3.7; *p* = 0.0315) and WLG scores (18.5 ± 7.4 vs. 23.9 ± 6.3; *p* = 0.0462) than those with LCLs but lacking ICLs (Supplementary Fig. 1).

### Correlations between neuropsychological findings and clinical parameters

**Figure 1:**
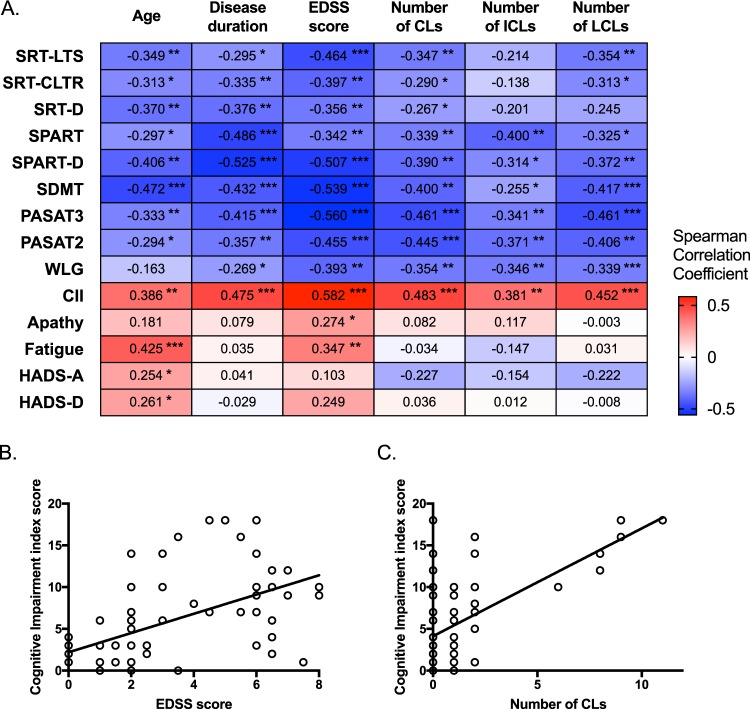
Correlation between neuropsychological test results and clinical parameters in MS patients. (**A**) Heatmap displays Spearman correlation coefficients between neuropsychological test results and clinical parameters. * *p* < 0.05, ** *p* < 0.01, *** *p* < 0.001. (**B**) Correlation between CII and EDSS scores. (**C**) Correlation between CII scores and the number of CLs. AS, Apathy Scale; CII, Cognitive Impairment Index; CLs, cortical lesions; EDSS, Expanded Disability Status Scale; FQ, Fatigue Questionnaire; HADS, Hospital Anxiety and Depression Scale; HADS-A, anxiety score of the HADS; HADS-D, depression score of the HADS; ICLs, intracortical lesions; LCLs, leukocortical lesions; MS, multiple sclerosis; NS, not significant; PASAT, Paced Auditory Serial Addition Test; PASAT-2, 2-s version of the PASAT; PASAT-3, 3-s version of the PASAT; SDMT, Symbol Digit Modalities Test; SPART, 10/36-Spatial Recall Test; SPART-D, delayed recall of the SPART; SRT, Selective Reminding Test; SRT-LTS, long-term storage of the SRT; SRT-CLTR, consistent long-term retrieval of the SRT; WLG, word list generation test.

We next examined the correlations between the results of neuropsychological examinations and the following variables: age at examination, disease duration, EDSS score, the number of CLs, the number of ICLs, and the number of LCLs. All tests of BRB-N were negatively correlated with age at examination, disease duration, EDSS score, and the number of CLs, except for the WLG score with age at examination (Fig. 1A). CII scores were positively correlated with age at examination, disease duration, EDSS score, number of CLs, number of ICLs, and number of LCLs. AS scores were positively correlated only with the EDSS score (ρ = 0.27, *p* = 0.0329), whereas FQ scores were correlated with age at examination and with the EDSS score (ρ = 0.43, *p* = 0.0006 and ρ = 0.35, *p* = 0.0061, respectively). HADS-A and HADS-D scores were weakly associated with age at examination (ρ = 0.25, *p* = 0.0482 and ρ = 0.26, *p* = 0.0418, respectively). The associations of CII scores with EDSS scores and with the number of CLs were the most robust among all the comparisons (ρ = 0.58, *p* < 0.0001 and ρ = 0.48, *p* < 0.0001, respectively) (Fig. 1B,C).

### Comparison of neuropsychological assessment scores between carriers and non-carriers of HLA–DRB1*15:01 and HLA–DRB1*04:05

Neuropsychological status was compared between patients with and without the HLA–DRB1*15:01 or HLA–DRB1*04:05 alleles (Supplementary Table 2). There were no significant differences in the neuropsychological assessment scores between the carriers and the non-carriers of each of these alleles.

### Logistic regression analysis for potential confounders of effects on cognitive dysfunction

Univariate and multivariate regression analyses were performed to elucidate the factors contributing to poor cognitive function in Japanese MS patients (Table 3). Univariate regression analysis showed that age at examination, disease duration, clinical subtype of the disease (RRMS versus SPMS), EDSS score, and number of CLs, but not HLA–DRB1*15:01 or HLA–DRB1*04:05, were significantly associated with the highest quartile of CII score. With forward stepwise selection, the EDSS score and the number of CLs were included in the multivariate regression analysis, which demonstrated that both EDSS score and number of CLs were independent predictors of the highest quartile of CII scores in Japanese patients with MS (EDSS score: odds ratio 2.13, *p* = 0.0003; number of CLs: odds ratio 2.38, *p* = 0.0070).

**Table 3:** Logistic regression analysis for potential confounders of the highest quartile of Cognitive Impairment Index scores in MS patients.

	Univariate Analysis			Multivariate Analysis		
	Odds ratio	95% CI (Lower, Upper)	*p* value	Odds ratio	95% CI (Lower, Upper)	*p* value
Sex (female/male)	0.24	(0.01, 2.09)	0.2004	Omitted^†^		
Age at examination, years	1.14	(1.04, 1.28)	0.0019	Omitted^†^		
Disease duration, years	1.21	(1.07, 1.43)	0.0003	Omitted^†^		
RRMS/SPMS	0.03	(0.00, 0.21)	<0.0001	Omitted^†^		
HLA–DRB1*15:01	1.05	(0.25, 4.31)	NS	Omitted^††^		
HLA–DRB1*04:05	1.43	(0.61, 5.81)	NS	Omitted^†^		
EDSS score	2.42	(1.52, 4.90)	<0.0001	2.13	(1.36, 4.15)	0.0003
Number of CLs	2.48	(1.31, 7.45)	0.0002	2.38	(1.15, 11.14)	0.0070

^†^Omitted as a result of stepwise selection. CI, confidence interval; NS, not significant.

## Discussion

In the present study, SDMT, PASAT-2, and PASAT-3 scores were significantly lower in our MS patients compared with controls, indicating impairments of attention, information processing, and working memory. Additionally, lower SPART and SPART-D scores indicated visual memory impairment in Japanese MS patients, an aspect of cognition that is also commonly affected even during the early phase of the disease in Caucasian MS patients^1^. The lower SRT-LTS and WLG scores suggest impairment of verbal memory and verbal fluency in our MS patients, which is observed in around half of Caucasian MS patients^1^. In the present study, WLG score did not correlate with age. We believe this lack of correlation in part resulted from the age distribution of this cohort, which included patients from 16 to 72 years of age and lacked very elderly participants. Similarly, Niino *et al*. reported that WLG scores did not correlate with age in either 184 MS patients or in 163 healthy controls with a similar age distribution^6^. However, WLG negatively correlated with disease duration, EDSS score, and the number of CLs, ICLs, and LCLs, suggesting that verbal fluency was affected more profoundly by the disease course and disability progression of MS as well as the presence of CLs than by aging. These observations suggest the patterns of cognitive impairment in Japanese patients with MS are largely similar to those in Caucasian patients.

Although the prevalence and the number of CLs, ICLs, and LCLs were relatively low in our subjects compared with Caucasian MS patients, we found that the number of CLs in Japanese MS patients was significantly associated with all of the BRB-N tests. This result is in accord with previous studies of Caucasian MS patients^9,12,13^ and with an earlier study of Japanese MS patients, which reported an association between the presence of CLs and cognitive dysfunction^17^. The presence and number of CLs were strongly associated with the overall CII score in our study, which is also consistent with a previous study in Caucasian MS patients^21^. The results of multivariate regression analysis also indicate that both the number of CLs and the EDSS score are independent predictors of the most severe cognitive decline in Japanese MS patients. The presence and the number of CLs could therefore be relevant risk factors for cognitive impairment in Japanese patients with MS, even though Japanese patients have CLs less frequently than Caucasian patients.

As for the cognitive subdomains assessed, our study revealed that in Japanese MS patients, the presence and number of CLs were more significantly associated with scores in the SDMT, PASAT-3, and SPART-D than with SRT-LTS scores. This finding suggests that CLs contribute more to impairments of attention, information processing, and working and visual memory than verbal memory. A study of Caucasian MS patients also reported a more robust association of CL numbers with PASAT and SDMT scores than with SRT-LTS scores^21^. Therefore, CLs are more strongly related to the cognitive subdomains that are preferentially affected in the early phase of the disease^1^, which further suggests that CLs are an important risk factor for early cognitive impairment in MS.

Concerning the subtype of CLs, pathologically classified type I lesions correspond to LCLs, while type II–IV lesions correspond to ICLs^28^. However, type III subpial lesions are only detectable by 7-tesla high-resolution MRI, not conventional 3-tesla MRI^14^. LCLs were reported to affect total cognitive function more severely than ICLs, based on 3-tesla MRI of Caucasian MS patients^13^. A recent study using 7-tesla MRI demonstrated that type I CLs (LCLs) were most frequently associated with impairments in various domains of cognitive function, such as visuospatial, learning/memory, processing speed, and semantic language domains, while type III and IV CLs (ICLs) were associated with poor performance in learning/memory and semantic language domains^14^. In the present study, we found that LCLs in patients were more extensively associated with various domains of neuropsychological assessments than ICLs, although the effects on overall CII scores were comparable. However, ICLs, including in patients who had both ICLs and LCLs, were more significantly associated with lower scores in the SPART and WLG test than LCLs. This finding suggests that the presence of ICLs may relate to impairments of verbal and visual memory. Given the association of type III and IV CLs, which are included in the ICL category, with impairment of learning/memory and semantic language tests in Caucasian MS patients^14^, it is possible that ICLs may relate to impairments in somewhat distinct domains of cognitive functions from LCLs. This point should be clarified in future large-scale studies. Overall, although we could not assess type III subpial lesions by 3-tesla MRI, these results are roughly consistent with results from studies of Caucasian MS patients^13,14^.

In this study, we did not find any significant differences in the neuropsychological status of carriers and non-carriers of the HLA–DRB1*1501 and HLA–DRB1*04:05 alleles. Although we previously reported that HLA–DRB1*15:01 increased CLs, whereas HLA–DRB1*04:05 decreased CLs^16^, the MS-susceptible *HLA–DRB1* genotypes did not influence the neuropsychological outcome in the present study. CLs contribute to cognitive impairment, but such an impairment could be influenced by much broader damage to brain structures. For example, cognitive dysfunction is also associated with losses of volume in subcortical structures, such as the caudate^29^ and the thalamus^30^. Therefore, although the HLA alleles for MS susceptibility can facilitate CL development, they are not so influential as to modulate cognitive decline.

There are several limitations to the present study. First, the sample size was relatively small, mainly because of the low prevalence of MS in Japan^31^ and the difficulties in enrolling patients prospectively to undergo both MRI and neuropsychological studies within a 1-week period in a single institution. Second, because 3-tesla MRI was used, subpial lesions could not be assessed and evaluating which CLs extended into the subcortical WM was occasionally difficult, even though two examiners independently assessed the CLs. Third, the relationship between the location of CLs and cognitive functions was not assessed because of the small number of enrolled patients; this should be investigated in future studies. Fourth, volumetric measurement of WM, GM, and T2 lesions were lacking, because the present study was initially designed to determine whether CLs imaged with 3-tesla 3D-DIR MRI might be an indicator of cognitive impairment in real-world settings in the Japanese population. Thus, such volumetric analysis should be performed in future studies to further evaluate factors contributing to cognitive dysfunction.

Nonetheless, in this study we demonstrated the contribution of CLs to overall cognitive impairment in Japanese MS patients. LCLs were more significantly associated with the impairment of cognitive domains preferentially affected by MS, including attention, information processing, and working and visual memory, while ICLs may contribute more to verbal and visual memory impairments. The presence and number of CLs could be relevant risk factors for cognitive decline in Japanese MS patients.

## Materials and Methods

### Participants

Sixty-one patients with MS who gave written informed consent prior to their inclusion in this study were prospectively and consecutively enrolled in the period from February 1, 2016, to May 31, 2017 (Table 1). The diagnosis of MS was based on the established criteria at that time^32^. All patients were carefully followed-up at a single MS centre in the district, the Department of Neurology at Kyushu University Hospital. Patients were clinically stable with no relapse at least for 1 month prior to MRI (including 3D-DIR imaging) and neuropsychological assessment, which were both performed within a 1-week period. This study was approved by the Ethics Committee of Kyushu University and was conducted according to the World Medical Association Declaration of Helsinki.

### MR imaging and identification of CLs

**Figure 2:**
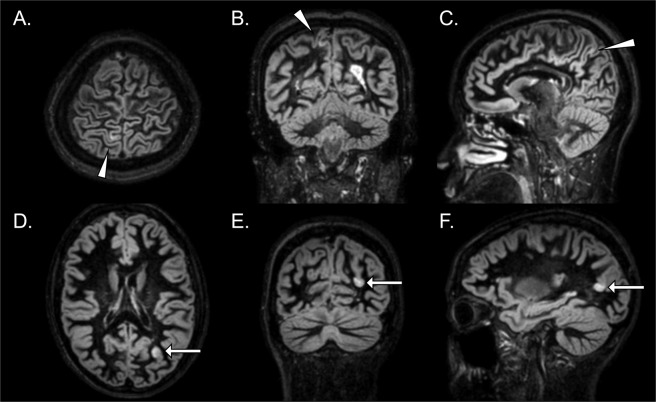
Examples of cortical lesions on 3-dimensional double inversion recovery (DIR) images from two patients with MS. (**A–C**) Arrowheads indicate an intracortical lesion that does not appear to extend into the subcortical white matter. (**D–F**) Arrows indicate a leukocortical lesion that involves both the grey and white matter.

All MRI images were obtained with a 3-tesla scanner (Achieva; Philips, Best, The Netherlands) with the following sequence parameters, as previously described^16^: repetition time (TR)/echo time (TE) = 5500/255–284 ms, inversion time (TI) = 450/2550 ms, echo train length (ETL) = 173, matrix size = 208 × 208, and field of view (FOV) = 250 mm × 250 mm. Lesions were identified based on consensus between two examiners, an experienced neuroradiologist (A.H.) and a neurologist (K.S.), with special attention to the recommendations made by Geurts *et al*.^33^. CLs were classified into ICLs localized in the cortical GM and LCLs encompassing both GM and WM (Fig. 2).

### Neuropsychological assessments

Participants underwent the Japanese version of BRB-N^6^, which comprises the following tests: the Selective Reminding Test (SRT) of verbal learning memory; the 10/36-Spatial Recall Test (SPART) of visuospatial memory and learning; the Paced Auditory Serial Addition Test (PASAT) and Symbol Digit Modalities Test (SDMT) of attention, information processing, and working memory; and the word list generation (WLG) test of verbal fluency. The battery of tests was performed in the following order: SRT, SPART, SDMT, PASAT, delayed recall of SRT (SRT-D), delayed recall of SPART (SPART-D), and WLG. These tests included the following variants: for the SRT, the long-term storage (SRT-LTS), consistent long-term retrieval (SRT-CLTR), and delayed recall (SRT-D) tests; for the SPART, the immediate recall (SPART) and delayed recall (SPART-D) tests; and for the PASAT, both the 2-s and 3-s versions (PASAT-2 and PASAT-3). Cognitive impairment index (CII) scores were computed by calculating the mean and the SD of the control data^6^, as previously described^34^. The results obtained from the controls in our previous study^6^ were used for comparison with the total MS patient group in this study. Apathy was assessed with the Apathy Scale (AS)^35^, using a translated version of the scale that has been previously validated in Japanese^36^. Fatigue was evaluated with the Japanese version of the Fatigue Questionnaire (FQ)^37^. Depression and anxiety were assessed with the Japanese version of the Hospital Anxiety and Depression Scale (HADS-A and HADS-D)^38^.

### HLA genotyping

The genotypes of the *HLA–DRB1* alleles were analysed in all patients by using hybridization between the polymerase chain reaction amplification products of the genes and the sequence-specific oligonucleotide probes, as previously described^39^.

### Statistics

Statistical analysis was performed with JMP Pro 12.2.0 (SAS Institute, Cary, NC, USA) or GraphPad Prism 7.0d (GraphPad Software, San Diego, CA, USA). Fisher’s exact test was used to compare categorical variables, and the Wilcoxon rank-sum test was used to analyse continuous scales. Correlations among continuous scales were calculated by using Spearman’s rank correlation coefficient. To perform the regression analysis, we divided the CII scores of patients into quartiles defined by the following ranges: 0–1, 2–4, 5–9, and ≥10. Univariate and multivariate logistic regression analysis was performed to assess possible factors associated with the lowest quartile of the CII score, including sex, age at examination, disease duration, Kurtzke Expanded Disability Severity Scale (EDSS) score, number of CLs, presence of the HLA–DRB1*15:01 allele, and presence of the HLA–DRB1*04:05 allele. Given the small sample size, forward stepwise selection was performed to reduce the number of predictors incorporated into the multivariate analysis^40^. A *p*-value < 0.05 was considered statistically significant.

## Supplementary information

Supplementary Information.
