# Supplementary material for: Contribution of cortical lesions to cognitive impairment in Japanese patients with multiple sclerosis
Source: Sci Rep. 2020 Mar 23;10:5228. doi: 10.1038/s41598-020-61012-3 (PMC7090088; doi:10.1038/s41598-020-61012-3)
Supplement: Supplementary file 1 — Supplementary Information. [file 41598_2020_61012_MOESM1_ESM.docx]

**Contribution of cortical lesions to cognitive but not psychiatric impairment in Japanese patients with multiple sclerosis**

Koji Shinoda^1^, Takuya Matsushita^1^, Yuri Nakamura^1^, Katsuhisa Masaki^1^, Shiori Sakai^1^, Haruka Nomiyama^1^, Osamu Togao^2^, Akio Hiwatashi^3^, Masaaki Niino^4^, Noriko Isobe^5^, and Jun-ichi Kira^1^

^1^Department of Neurology, Neurological Institute, Graduate School of Medical Sciences, Kyushu University, Fukuoka, Japan. ^2^Department of Clinical Radiology, Graduate School of Medical Sciences, Kyushu University, Fukuoka, Japan. ^3^Department of Molecular Imaging & Diagnosis, Graduate School of Medical Sciences, Kyushu University, Fukuoka, Japan. ^4^Department of Clinical Research, National Hospital Organization Hokkaido Medical Center, Sapporo, Japan. ^5^Department of Neurological Therapeutics, Neurological Institute, Graduate School of Medical Sciences, Kyushu University, Fukuoka, Japan.

**Supplementary Table 1.** Comparison of neuropsychological assessment results between healthy controls and MS patients. Data are shown as the mean (standard deviation).

|  | HCs (n = 115) | MS (n = 61) | *p* value |
| --- | --- | --- | --- |
| SRT-LTS | 50.1 (10.6) | 43.8 (16.7) | 0.0367 |
| SRT-CLTR | 43.4 (13.4) | 37.8 (18.4) | 0.0782 |
| SRT-D | 9.5 (1.9) | 8.9 (3.3) | NS |
| SPART | 23.0 (4.3) | 19.5 (5.3) | <0.0001 |
| SPART-D | 8.1 (1.9) | 7.2 (2.3) | 0.0169 |
| SDMT | 63.1 (10.6) | 44.1 (14.9) | <0.0001 |
| PASAT-3 | 52.4 (6.8) | 42.0 (12.6) | <0.0001 |
| PASAT-2 | 40.9 (8.5) | 30.4 (10.6) | <0.0001 |
| WLG | 29.0 (4.9) | 23.1 (6.9) | <0.0001 |

**Supplementary Table 2.** Comparison of neuropsychological tests between carriers and non-carriers of the *HLA-DRB1*15:01* or *HLA-DRB1*04:05* alleles*.* Data are shown as the mean (standard deviation).

|  | ***HLA-DRB1*15:01*** | | | ***HLA-DRB1*04:05*** | | |
| --- | --- | --- | --- | --- | --- | --- |
|  | **Carrier**  **(n = 20)** | **Non-carrier**  **(n = 41)** | *p* value | **Carrier**  **(n = 26)** | **Non-carrier**  **(n = 35)** | *p* value |
| SRT-LTS | 40.2 (19.3) | 45.6 (15.2) | NS | 43.5 (17.3) | 43.5 (16.5) | NS |
| SRT-CLTR | 34.6 (20.2) | 39.3 (17. 5) | NS | 38.1 (19.5) | 38.1 (17.8) | NS |
| SRT-D | 8.4 (4.0) | 9.2 (2.9) | NS | 9.0 (3.4) | 9.0 (3.3) | NS |
| SPART | 19.8 (5.8) | 19.4 (5.1) | NS | 19.3 (5.2) | 19.3 (5.5) | NS |
| SPART-D | 7.0 (2.4) | 7.3 (2.3) | NS | 7.4 (2.3) | 7.4 (2.4) | NS |
| SDMT | 45.0 (19.0) | 43.7 (12.6) | NS | 43.1 (14.9) | 43.1 (15.0) | NS |
| PASAT-3 | 41.6 (14.4) | 42.3 (11.8) | NS | 40.7 (12.7) | 40.7 (12.6) | NS |
| PASAT-2 | 31.4 (13.1) | 29.9 (9.2) | NS | 29.5 (9.4) | 29.5 (11.5) | NS |
| WLG | 23.1 (7.5) | 23.2 (6.7) | NS | 22.7 (7.3) | 22.7 (6.7) | NS |
| CII | 6.5 (6.3) | 5.5 (4.7) | NS | 62 (5.6) | 6.2 (5.1) | NS |
| AS | 16.0 (6.5) | 15.8 (7.8) | NS | 16.1 (7.9) | 16.1 (7.0) | NS |
| FQ | 115.5 (32.7) | 128.3 (29.3) | NS | 129.3 (31.2) | 129.3 (30.3) | NS |
| HADS-A | 7.0 (2.8) | 7.2 (4.5) | NS | 7.7 (4.7) | 7.7 (3.5) | NS |
| HADS-D | 7.3 (3.9) | 7.0 (4.7) | NS | 6.4 (4.0) | 6.4 (4.7) | NS |

**Supplementary Figure 1. Comparison of neuropsychological test results according to the presence or absence of ICLs among MS patients with CLs.**

Comparisons of neuropsychological test results between MS patients with ICLs (with or without LCLs) and patients with only LCLs (without ICLs) were performed by using the Wilcoxon rank-sum test. CII, Cognitive Impairment Index; CL, cortical lesion; HADS, Hospital Anxiety and Depression Scale; HADS-A, anxiety score of the HADS; HADS-D, depression score of the HADS; NS, not significant; ICL, intracortical lesions; LCL, leukocortical lesions; PASAT, Paced Auditory Serial Addition Test; PASAT-2, 2-s version of PASAT; PASAT-3, 3-s version of PASAT; SDMT, Symbol Digit Modalities Test; SPART, 10/36-Spatial Recall Test; SPART-D, delayed recall of the SPART; SRT, Selective Reminding Test; SRT-LTS, long-term storage of the SRT; SRT-CLTR, consistent long-term retrieval of the SRT; WLG, word list generation test.

**
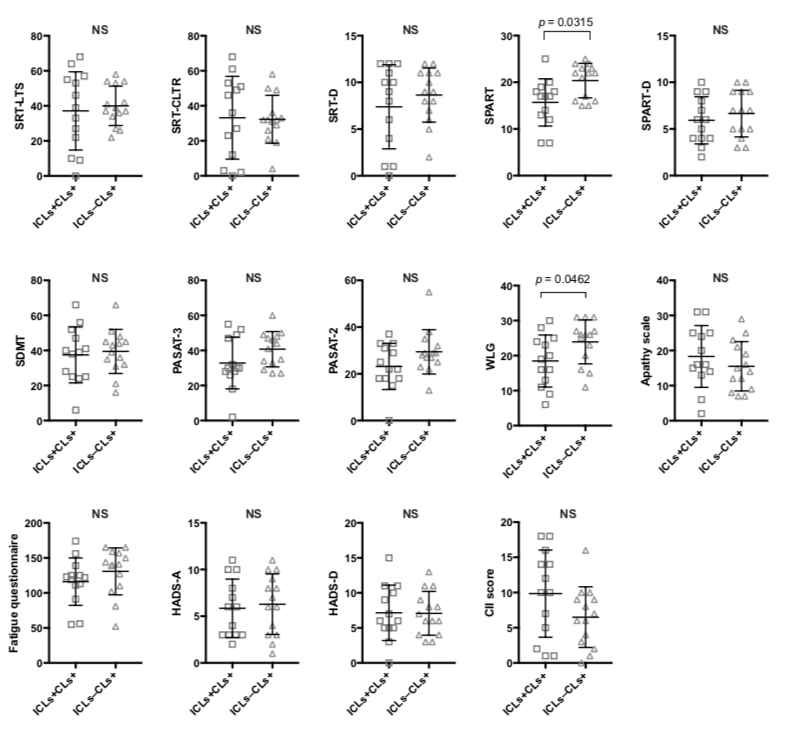
**
